# Innate Immunity in Insects: The Lights and Shadows of Phenoloxidase System Activation

**DOI:** 10.3390/ijms26031320

**Published:** 2025-02-04

**Authors:** Agnieszka Zdybicka-Barabas, Sylwia Stączek, Magdalena Kunat-Budzyńska, Małgorzata Cytryńska

**Affiliations:** Department of Immunobiology, Institute of Biological Sciences, Faculty of Biology and Biotechnology, Maria Curie-Skłodowska University, Akademicka 19 St., 20-033 Lublin, Poland; agnieszka.zdybicka-barabas@mail.umcs.pl (A.Z.-B.); sylwia.staczek@mail.umcs.pl (S.S.); magdalena.kunat-budzynska@mail.umcs.pl (M.K.-B.)

**Keywords:** entomopathogenic fungi, melanization, parasitoid wasps, phenoloxidase system, proPO activation

## Abstract

Melanogenesis and melanin deposition are processes essential for the effective immune response of insects to various invaders. Phenoloxidase (PO), produced in specialized cells as an inactive precursor prophenoloxidase (proPO), is the key enzyme for melanin formation. The precursor is activated via limited proteolysis by a dedicated serine proteinase, which is the final element in the cascade of serine proteinases (SPs) that make up the PO system. Melanogenesis provides different cytotoxic molecules active in fighting infections, as well as melanin, which is important for sequestration of invaders. However, since the cytotoxic reactive compounds generated during melanization also pose a threat to host cells, strict control of the PO system is necessary for host self-protection. Different pathogens and parasites influence the PO system and melanization through various strategies, which allow them to survive and develop in the host insect body. In this review, we characterize “the lights and shadows” of PO system activation, indicating, on one hand, its advantages as an efficient and effective mechanism of the insect immune response and, on the other hand, the dangers for the insect host associated with the improper functioning of this system and selected strategies for regulating its activity by entomopathogenic organisms.

## 1. Introduction

**Figure 1:**
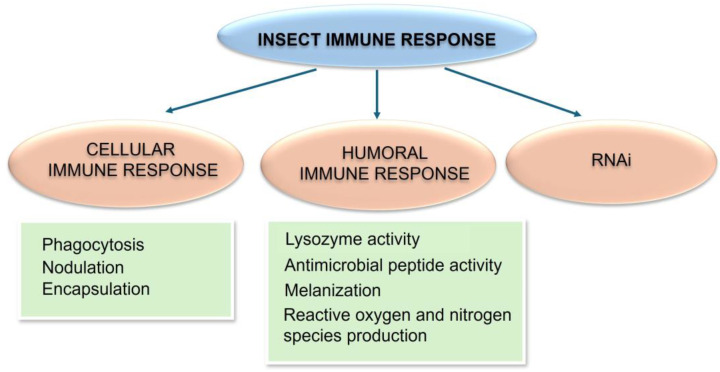
Insect innate immune mechanisms activated in response to different pathogens.

Insect immunity relies on innate mechanisms, which include cellular and humoral reactions in response to pathogen infections and parasite infestations. The cellular immune response depends on hemocytes, which are responsible for phagocytosis, nodulation, and encapsulation processes, while the humoral immune response involves various soluble factors and consists of (i) activation of the phenoloxidase (PO) system, leading to melanization, (ii) activation of signaling pathways, e.g., Toll, Imd, and JAK/STAT pathways, leading to synthesis of antimicrobial peptides (AMPs) and other immune-related factors, and (iii) coagulation of hemolymph, resulting in cloth formation engaged in wound healing [1,2,3,4,5,6]. All these mechanisms are interconnected and cooperate not only to fight pathogens and parasites but also to maintain the integrity of the organism (homeostasis) related to, e.g., the preservation of the symbiotic microbiota [1,7,8] (Figure 1).

The initiation of immune mechanisms in response to infection requires proper recognition of pathogens, which is based on the interaction of pathogen-associated molecular patterns (PAMPs) with appropriate pathogen recognition receptors (PRRs). This initial step is decisive for the subsequent steps of the immune response. The insect immune system can recognize components of microbial cell walls, e.g., bacterial lipopolysaccharide (LPS), peptidoglycan (PGN), and lipoteichoic acid (LTA), as well as fungal β-1,3-glucans. Moreover, insects can distinguish between lysine-type PGN (Lys-PGN) and *meso*-diaminopimelic-type PGN (Dap-PGN) of Gram-positive and Gram-negative bacteria, respectively [9,10]. In addition, it has recently been demonstrated that the immune system of the greater wax moth *Galleria mellonella* recognizes fungal α-1,3-glucan, indicating this molecule as a novel PAMP [11,12,13]. 

The recognition of PAMPs requires interaction with proper PRRs. In general, in insects, peptidoglycan recognition proteins (PGRPs) are involved in PGN recognition, whereas Gram-negative bacteria-binding proteins (GNBPs)/β-1,3-glucan-binding proteins (β-GBPs) can recognize LPS and β-1,3-glucans, and some also recognize LTAs [2,14,15]. The β-GBPs/GNBPs are also known as LPS- and glucan-binding proteins (LGBPs). Other proteins, e.g., C-type lectins (CTLs), thioester-containing proteins (TEPs), scavenger receptors, apolipophorin III (apoLp-III), and hemolin, are also engaged in PAMP recognition in different insect species [16,17,18,19]. It should be mentioned that, in order to trigger immune reactions, the interaction of one type of PRRs with PAMPs is not always sufficient. The cooperation of different types of PRRs or the formation of "attack complexes" together with other immune effector molecules is sometimes necessary, as demonstrated for PGRP-SA and GNBP1, involved in Toll pathway activation, and for GNBP3, serpin Necrotic, and phenoloxidase during antifungal response in *Drosophila melanogaster* [20,21,22].

Insect hemocytes can be classified into different types depending on cell morphology, functional properties, expression of marker genes, and selected antigens recognized by monoclonal antibodies [23,24,25,26,27,28,29,30]. In lepidopteran species, hemocytes are usually divided into prohemocytes, plasmatocytes, granulocytes, spherulocytes, and oenocytoids. In dipteran species, e.g., *D. melanogaster*, prohemocytes, plasmatocytes, crystal cells, and lamellocytes are distinguished. Among them, plasmatocytes, granulocytes, and lamellocytes are adherent cells involved in the processes of cellular immune response, i.e., nodulation and encapsulation. Of note, *Drosophila* plasmatocytes, lamellocytes, and crystal cells functionally correspond to granulocytes, plasmatocytes, and oenocytoids, respectively, of lepidopteran species. In the context of melanization, oenocytoids, crystal cells, and lamellocytes can synthesize, store, and release the precursors of PO system components. 

Research on the melanization process and the role of phenoloxidase in insect physiology, including immune response, has been conducted in species belonging to different orders, e.g., Blattodea (*Periplaneta americana*), Coleoptera (*Tenebrio molitor* and *Tribolium castaneum*), Diptera (*Anopheles gambiae*, *Drosophila melanogaster*, and *Musca domestica*), Hemiptera (*Acyrthosiphon pisum*), Hymenoptera (*Apis mellifera* and *Bombus terrestris*), Lepidoptera (*Bombyx mori*, *Galleria mellonella*, *Hyalophora cecropia*, and *Manduca sexta*), Odonata (*Argia anceps*), and Orthoptera (*Acheta domesticus* and *Locusta migratoria*) [31]. Most studies on the participation of PO in insect immunity have been conducted on representatives of the order Lepidoptera, while more detailed information on serine protease cascades and the activation and regulation of the PO system has been provided by studies on *D. melanogaster*, *M. sexta*, *B. mori*, *T. molitor*, and mosquitoes.

## 2. Phenoloxidase System in Insect Immunity

**Figure 2:**
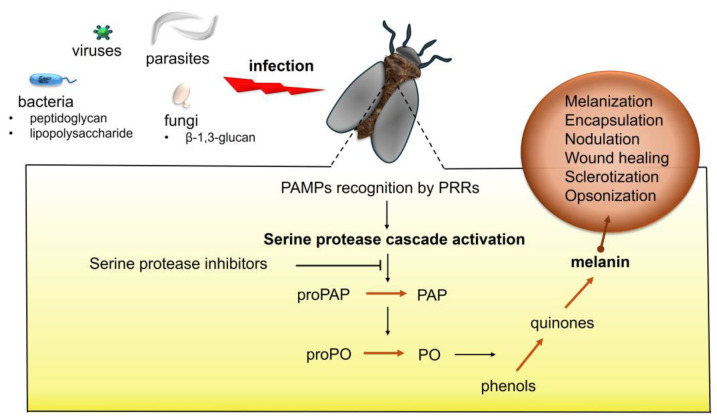
Simplified scheme of the activation and role of the PO system in insect immunity. Recognition of PAMPs by the host PRRs activates the serine proteinase cascade. The final stage is the activation of the zymogen of proPO-activating serine protease (proPAP) into active proPO-activating serine protease (PAP). Active PAP converts the proPO into functional PO. PO initiates the process of melanogenesis. Melanin is important for immune defense and physiological processes in the insect body.

Melanization is one of the humoral immune processes aimed at fighting pathogens and parasites. It is initiated by the activation of the PO system in the insect hemocoel and at the site of injury. The activation of the PO system results in formation of melanin from low-molecular-weight aromatic compounds (e.g., tyrosine) through a series of enzymatic and non-enzymatic reactions [24] (Figure 2). Melanization is considered one of the most rapid immune responses in insects. For example, in *D. melanogaster*, melanin can be formed at the site of injury as quickly as 10 min after treatment [32].

### 2.1. Synthesis and Release of Prophenoloxidase

The key enzyme responsible for the catalysis of the reaction initiating melanin formation is phenoloxidase, produced as an inactive precursor, i.e., prophenoloxidase (proPO). Insect POs belong to type-3 copper-containing proteins, similarly to arthropod and molluscan hemocyanins and mammalian tyrosinases [22,31,32,33,34]. They contain two copper ions, each coordinated by three conserved histidine residues. Two types of POs have been described in insects: (i) enzymes with tyrosinase-like activities known to be involved in the immune response, which are able to catalyze the hydroxylation of monophenols (e.g., tyrosine) to *o*-diphenols and then the oxidation of *o*-diphenols to corresponding *o*-quinones, and (ii) laccases, which oxidize *o*- and *p*-diphenols to corresponding quinones and are responsible for sclerotization and tanning of the exoskeleton [35,36,37].

Insect proPOs can form homodimers, e.g., PPO8 from A. gambiae [38], or heterodimers, e.g., proPO1 and proPO2 from M. sexta or B. mori [39,40]. The monomers, with molecular weights of 70–80 kDa, are composed of three domains: (i) an N-terminal domain, (ii) a central α-helical domain containing the active site-binding copper ions, and (iii) a C-terminal domain with a β-sheet structure. The N-terminal domain is preceded by a pro-region, which contains a cleavage site for a dedicated trypsin-like serine protease (see Section 2.2) [39,41,42,43,44,45].

Insect genomes differ in terms of a number of genes encoding proPOs [35]. Only one gene coding for proPO has been characterized in *A. mellifera* (*AmproPO*) [46], three in *D. melanogaster* (*DmelPPO1-DmelPPO3*) [47], nine in *A. gambiae* (*AgPPO1-AgPPO9*) and *Culex quinquefasciatus* (*CqPPO1-CqPPO9*), and ten in *Aedes aegypti* (*AaPPO1-AaPPO10*) and *Aedes albopictus* (*AalPPO1-AalPPO10*) [48,49,50,51,52]. In the pea aphid *A. pisum*, in which the PO system and melanization play an essential role in immune response due to the incomplete immune system in comparison with other insect species, two proPO-encoding genes (*proPO1*, *proPO2*) have been described with homology to *D. melanogaster PPO3* [53,54].

The proenzyme is synthesized in different types of hemocytes and released to the hemolymph as a result of cell lysis [33,55,56,57]. In lepidopteran species, it is produced mainly in oenocytoids [35]; however, in *B. mori*, proPO was also detected in some granulocytes and plasmatocytes [55]. Interestingly, in *B. mori*, proPO can be transported from the hemolymph to the cuticle. It was shown that cuticular proPO, unlike proPO in the hemolymph, contains surface-exposed oxidized methionine residues. It is possible that this modification is relevant to the transepithelial transport of proPO from the hemolymph to the cuticle [40,41]. In some species in the order Diptera, e.g., *D. melanogaster*, crystal cells are responsible for PPO1 and PPO2 synthesis, while PPO3 is produced in lamellocytes. The two proPOs synthesized in the crystal cells provide PO activity in hemolymph after septic and aseptic injury, with PPO1 released rapidly and PPO2 contained in the form of crystals, which plays a role in the later phase of immune response [32,35,58,59,60]. Recently, a novel role of crystal cells and proPO in *Drosophila* has been demonstrated to be connected with reversible oxygen collection, which is dependent on the phase transition of PPO2 between the oxygenated crystalline form (oxygen reservoir) and the deoxygenated dissolved form supporting respiration [61]. On the other hand, *Drosophila* PPO3 is involved in melanin deposition during encapsulation after being released from lamellocytes, and this proenzyme does not require limited proteolysis for activation [32,62,63,64]. In another member of the order Diptera, the mosquito *A. gambiae*, oenocytoids and granulocytes can synthesize proPOs; however, PPO6 is expressed in all hemocytes [65,66,67], PPO2, PPO4, PPO5, and PPO9 are most abundant in granulocyte populations [49,65,67], and PPO1, PPO3, and PPO8 are enriched in oenocytoids [68,69,70]. 

It is still intriguing that proPOs are synthesized in hemocytes without a signal peptide, which implies release of the proenzymes via cell lysis, as indicated in lepidopteran species, e.g., *B. mori* [71], *M. sexta* [72], and *Spodoptera exigua* [73,74], and in mosquitoes, e.g., *A. gambiae* [70]. Recently, using *Drosophila* proPOs fused with signal peptides of different proteins, it has been demonstrated that the presence of a signal peptide can result in PPO glycosylation in vitro and in vivo. Glycosylated proPOs were secreted by the cells but they were not activated, probably due to conformational changes induced by this modification. It was concluded that the lack of a signal peptide prevents glycosylation of proPO, allowing it to maintain the appropriate conformation and activity [75]. There is some evidence suggesting that the release of insect proPOs may be controlled by the JNK pathway and calcium signaling [57,75,76]. It has also been reported that oenocytoid lysis is mediated by prostaglandin E2 [70,73].

### 2.2. ProPO Activation—Serine Proteinase Cascades

In general, the activation of proPO occurs via limited proteolysis by a dedicated serine proteinase, which is the final element in the cascade of serine proteinases (SPs) that make up the PO system. The enzymes directly involved in proPO activation have different names, e.g., proPO-activating enzyme (proPAE/PPAE), proPO-activating proteinase (proPAP/PAP), and proPO-activating factor (proPAF/PPAF) in *B. mori* [77], *M. sexta* [78,79,80], and the Korean black chafer *Holotrichia diomphalia* (Coleoptera), respectively [81,82]. The activated proteinase cleaves proPO in its N-terminal region at approximately the 50th residue at a conserved Arg-Phe (or in some cases Arg-Val) bond, which results in proPO activation [77,78,79,80,81,82]. However, the pathway leading to proPO activation is complex and, depending on the insect species as well as the PAMPs recognized, both the number of serine proteinases and the course of activation of proPO to PO may be different. In addition, other components can also be involved in the PO system, e.g., serine proteinase homologs (SPHs) devoid of proteolytic activity and serine proteinase inhibitors (e.g., serpins) [20,24,83,84]. Interestingly, the serine proteinases as well as SPHs contain one or several clip domains in their pro-regions [85].

**Figure 3:**
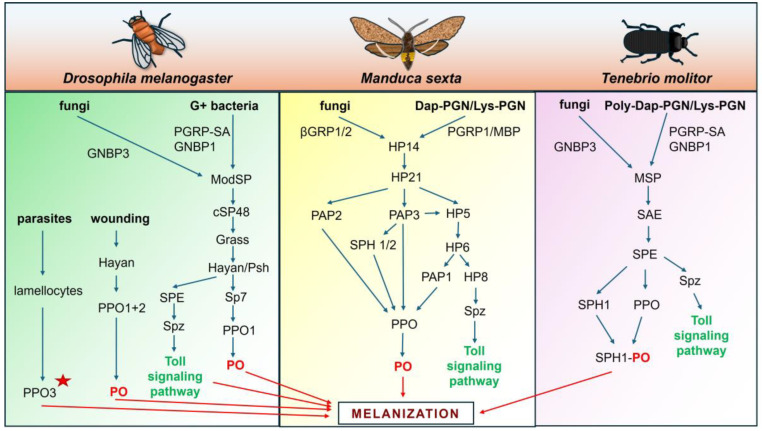
Serine proteinase cascades involved in proPO activation in different insect species. Interaction of PRRs with PAMPs leads to the activation of modular serine proteinases, which then trigger the activation of SPs and SPHs in cascades that make up the PO systems. This results eventually in the activation of proPO to enzymatically active PO. *D. melanogaster* PPO3 does not require proteolytic activation, as marked by a red asterisk. As indicated, the cascades leading to the activation of the Toll pathway and melanization can share several serine proteinases. Further details can be found in the text.

After the interaction of proper PRRs, e.g., C-type lectins, PGRPs, β-GRPs, or LGRPs, with bacterial or fungal surface PAMPs, autoactivation of the zymogen of the first serine proteinase in the cascade, i.e., an initiator proteinase, takes place. This most upstream proteinase is a modular proteinase with structural motifs similar to the mannose-associated serine proteinase (MASP) that initiates the complement system in vertebrates, e.g., ModSP in *D. melanogaster*, MSP in *T. molitor*, or hemolymph proteinase 14 (HP14) in *M. sexta* [22]. As shown schematically in Figure 3, the activated initiator proteinase triggers a cascade of clip-domain serine proteinases by the activation of the zymogen of the second proteinase.

In lepidopterans, e.g., *M. sexta*, the first zymogen proHP14 undergoes autoactivation in response to the formation of a multimeric complex composed of β-GRPs attached to β-1,3-glucans on the surface of fungal cells [83]. In addition, a complex of bacterial Lys- or Dap-type PGN with PGRP1 and MBP (microbe-binding protein; an ortholog of *Drosophila* GNBP1) also results in autoactivation of proHP14 [86,87]. Then, HP14 activates proHP21, which, after activation, can activate two proPAPs (proPAP2 and proPAP3). The activation of proPO by PAPs requires the presence of SPHs, e.g., SPH1 and SPH2. In addition, as presented in Figure 3, active HP21 and PAP3 in *M. sexta* can activate the zymogen of another clip-domain serine proteinase, i.e., proHP5. Next, HP5 cleaves proHP6, leading to the activation of proHP8 and proPAP1. Active PAP1 cleaves proPO, resulting in initiation of melanization, whereas active HP8 activates proSpätzle to Spätzle, leading to the activation of the Toll signaling pathway [88,89,90].

As also shown in Figure 3, in the dipteran *D. melanogaster*, the recognition of bacterial Lys-type PGN (but not Dap-type PGN) by PGRP-SA/GNBP1 or fungal β-1,3-glucan by GNBP3 leads to autoactivation of the most upstream serine proteinase ModSP, which triggers proteinase cascades, resulting in the activation of the Toll pathway and the PO system and melanization [20,91]. However, the activation of the Toll pathway occurs via the ModSP-cSP48-Grass-Persephone/Hayan-SPE (Spätzle-processing enzyme) cascade, whereas proPO activation requires another SP, namely Sp7, which is activated by Grass. It was demonstrated that Sp7 triggers the PO system and clearance of *Staphylococcus aureus*, however without melanin deposition. In addition, the activation of an SP named Hayan induces the melanization response at the site of injury and is involved in the blackening of wound sites. While Sp7 activation resulted in PPO1 activation in hemolymph, activated Hayan led to PPO1 and PPO2 activation at the site of injury, indicating separation of the PO system activation pathways in response to injury and infection [92].

Unlike in *M. sexta* and *D. melanogaster*, in the coleopteran species *T. molitor*, a single three-step serine proteinase cascade leads to proPO activation as well as activation of Toll signaling after recognition of bacterial PGN or fungal β-1,3-glucan [93,94,95,96,97]. As indicated in Figure 3, binding of Lys-type PGN by *Tm*PGRP-SA and *Tm*GNBP1 resulted in the formation of a complex with a zymogen of *Tm*MSP (proMSP) in which, in the presence of calcium ions, the activation of proMSP to MSP occurred. Active *Tm*MSP can convert a zymogen of *Tm*SPE-activating enzyme (proSAE) to active SAE, which is then able to convert a zymogen of *Tm*SPE to active SPE. Then, active *Tm*SPE can cleave a proSpätzle to an active cytokine Spätzle, which initiates Toll signaling. Moreover, *Tm*SPE can also cleave proPO and SPH1. This results in formation of a stable melanization complex which can induce local melanogenesis on the surface of microbial cells [93]. Interestingly, in *T. molitor*, similarly to *M. sexta* but unlike *D. melanogaster*, the activation of the initiator modular serine proteinase after recognition of Lys-type and Dap-type PGN leads to the activation of the PO system and Toll signaling [86,87,97]. 

The activation of proPO to PO initiates a process of melanogenesis involved in immune response. Phenoloxidase is responsible for catalyzing the first step in this process, i.e., hydroxylation of tyrosine to (3-(3,4-dihydroxyphenyl)alanine) (DOPA) and then its oxidation to Dopaquinone (*o*-quinone), which is converted to Dopachrome in a non-enzymatic reaction that occurs spontaneously. This compound undergoes decarboxylation, resulting in the formation of 5,6-dihydroxyindole (DHI), which is then oxidized by PO to form indole-5,6-quinone. Finally, indole-5,6-quinone polymerizes to black-brown eumelanin or, in the presence of cysteine or glutathione thiol groups, to yellow-red sulfur-containing pheomelanin. Phenoloxidase is also involved in other steps of melanogenesis, e.g., oxidation of Dopamine to Dopaminequinone [24,44,98].

### 2.3. Role of the PO System in Immunity

The process of melanogenesis initiated by PO has several important functions in insect immunity: (i) it is a source of a number of reactive intermediate products with cytotoxic properties towards pathogens and parasites (quinones and DHI), (ii) it supplies reactive oxygen and nitrogen species (ROS/RNS) supporting cytotoxic activity, (iii) the final product, i.e., melanin, sequesters pathogens and parasites by creating unfavorable conditions for the intruders, which leads to their death, (iv) cooperating with the coagulation system, it prevents the spread of pathogens and causes the killing of pathogens retained in the clot, (v) together with the coagulation system and with the participation of hemocytes, it facilitates wound healing and protects against the penetration of pathogens, and (vi) it supports cellular response reactions (nodulation and encapsulation) in the sequestration and neutralization of pathogens and parasites. The melanization process usually occurs locally, at the site of injury, while during nodulation and encapsulation, melanin is deposited directly on the surface of the intruders [12,31,92,99,100,101,102,103,104].

Nodulation and encapsulation are processes involving sequestration of pathogens (nodulation) or larger parasites (encapsulation) in multilayered structures made of hemocytes, called nodules or capsules, respectively. Lepidopteran species rely on plasmatocytes and granulocytes, whereas *Drosophila* relies on plasmatocytes and lamellocytes in these processes [24,101,105]. During nodulation, hemocytes recruited to the site of infection aggregate, which is accompanied by degranulation of granulocytes, leading finally to entrapment of bacterial cells or fungal spores in extracellular material. Plasmatocytes may then spread out over the surface of the aggregates, and additional melanin deposition may occur. The regulation and control of nodulation are carried out by eicosanoids, cytokines (e.g., plasmatocyte-spreading peptide, PSP), and components of the PO system. The microbicidal activity of intermediate products of melanin formation and ROS/RNS contributes to killing the pathogens [101,102,106,107]. On the other hand, encapsulation applies to larger foreign objects, such as parasitic protozoa and nematodes or eggs of parasitic wasps. Capsule formation proceeds with the involvement of granulocytes and plasmatocytes in lepidopteran species and plasmatocytes and lamellocytes in *Drosophila*. This process can occur with melanin deposition on the surface of an encapsulated object, in which PO activity is necessary. Crystal cells and lamellocytes in *Drosophila* but oenocytoids in other insects constitute the source of this enzyme [24,101,108].

**Figure 4:**
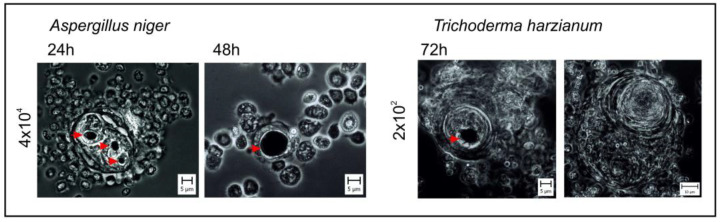
Images of nodules formed in the hemolymph of *Galleria mellonella* larvae after infection with fungal conidia. The larvae were immunized with different doses of *Aspergillus niger* and *Trichoderma harzianum* conidia. The hemolymph samples were obtained at different time points after infection. Nodules were imaged using a contrast-phase microscope OLYMPUS BH2. Red arrows indicate melanin deposition in the nodules.

As mentioned above, the mechanisms of humoral and cellular immune response cooperate to better protect the organism against development of infections. An example of such cooperation is the process of melanization (and also coagulation) associated with cellular reactions, i.e., nodulation and encapsulation. Melanization occurring in nodules and capsules contributes to the neutralization or killing of the intruders inside these structures (Figure 4). Direct evidence of the in vivo antimicrobial action of melanin has been recently provided by research on *G. mellonella* larvae infected with the fungus *Cryptococcus neoformans* [109]. In this interesting study, *C. neoformans* death in melanized nodules formed in vivo was visualized using an endogenously expressed GFP-based fungal viability assay. *G. mellonella* larvae were infected with a GFP-expressing strain of *C. neoformans*, which showed loss of a fluorescence signal upon cell death. In addition, this study also indicated the influence of *Candida albicans* and *Candida auris* morphological switch on the effectiveness of melanization, showing that yeast-like cells, in contrast to hyphae and pseudohyphae, induced a fast melanization reaction [109].

### 2.4. Regulation of PO System Activity

The activation of the PO system and the melanization process play an essential role in insect immune response, as they are engaged in fighting many pathogens and parasites. The main weapons in this fight are cytotoxic intermediate products as well as ROS and RNS generated during melanogenesis. Although they are necessary for fast and effective killing of invaders, they concurrently pose a threat to the cells in the host organism. This is one of the reasons why the melanization process takes place locally, and the hemocytes surrounding the foreign body on whose surface melanin is deposited also constitute a barrier for these cytotoxic molecules and free radicals, which therefore do not escape from the formed nodules and capsules. In order to prevent undesirable effects of its activation, the PO system must be subjected to strict control and regulation. This applies to the regulation of the activity of serine proteinases that are components of the PO system cascade and to the activation of proPO and the activity of PO itself.

The inhibitors of these proteinases are important factors regulating serine proteinase cascades. Among them, there are primarily inhibitors belonging to the serpin superfamily. Members of this widely distributed protein superfamily contain three β-sheets, 7–9 α-helices, and an exposed reactive center loop (RCL) at the C-terminus, determining their activity and specificity. Most serpins irreversibly inhibit the target enzymes by formation of a covalently bound enzyme–serpin complex. This is connected with rapid conformational changes in the serpin molecule, where the hinge region of RCL inserts into β-sheets, eventually resulting in distortion and inactivation of the enzyme active site [110,111,112]. A total of 32 and 34 genes encoding serpins have been identified in *M. sexta* and *B. mori*, respectively, among which 17 are orthologous pairs [113,114]. Similarly, many genes coding for serpins have been identified in other insect species: 31 in the mosquito *C. quinquefasciatus* and the beetle *T. castaneum*, 29 in *D. melanogaster*, 23 in *A. aegypti*, and 18 in *A. gambiae*. On the other hand, only seven serpin genes were identified in *A. mellifera* [112]. In many cases, involvement of serpins in the control of the PO system proteinase cascade has been demonstrated, and they are the most important factors regulating the activity of this system [91,115,116,117,118,119,120,121,122,123].

In addition to serpins, Kunitz-type proteinase inhibitors have been described as negative regulators of the PO system in *B. mori* and *M. sexta* [124]. Inhibitors of this type are ubiquitous in natural systems, serving, among others, as protease inhibitors in host defense against microbial infections or as ion channel blockers in venoms of poisonous animals. They contain a characteristic cysteine-rich Kunitz motif (~60 amino acid residues) with α-helix and β-sheet structures stabilized by three conserved disulfide bridges [125,126]. Kazal-type serine proteinase inhibitors also play important roles in many processes, including innate immunity [127,128], and can be involved in negative regulation of the PO system. Recently, a novel Kazal-type serine protease inhibitor, named AsKSPI, has been identified in the Indian moon moth *Actias selene* and characterized as a melanization inhibitor in *A. selene* hemolymph. AsKSPI inhibited proPO activation at the initial stage but did not affect PO activity after proPO was already activated [129]. Furthermore, it has recently been reported that a QM protein, i.e., a ribosomal protein L10 homolog, known to have extraribosomal functions in eukaryotic cells, is engaged in negative regulation of melanization in *B. mori*. Recombinant *BmQM* protein inhibited proPO activation in *B. mori* hemolymph, which resulted in inhibition of systemic melanization in *M. luteus*-injected larvae. On the other hand, *BmQM* suppression led to a considerable increase in PO activity [130]. The involvement of the *QM* gene and protein in immune response, including regulation of PO activity, was demonstrated in some invertebrates, e.g., shrimps *Litopenaeus vannamei*, *Penaeus japonicus*, and *Penaeus monodon* [131,132,133]. Although the mechanism of this regulation in insects and other invertebrates has not been elucidated yet, it is possible that QM proteins can be involved in the regulation of gene expression due to their ability to bind to the nuclear transactivator c-Jun, one of the components of the transcription factor Activator protein 1 (AP-1) complex. Interestingly, the involvement of AP-1 in the positive regulation of melanization of *Dirofilaria immitis* microfilariae was shown in the mosquito *Armigeres subalbatus* [134]. In addition, as demonstrated using far-Western blotting, *BmQM* was able to interact with *Bm*Jun in vitro [130]. 

Another level of melanization control is connected with PO enzymatic activity. Active PO can be inhibited by competitive peptide inhibitors containing DOPA in their sequence. Such peptide inhibitors were discovered in some insect species. In *Musca domestica*, the phenoloxidase inhibitor (POI) is composed of 38 amino acid residues, including DOPA in position 32, which is responsible for direct inhibition of PO activity [135]. On the basis of homology with *M. domestica* POI, a putative melanization inhibitor was cloned and characterized in *A. gambiae*. Gene knockdown resulted in more extensive melanization at the site of injury [136]. In turn, a 43 kDa melanization-inhibitory protein (MIP) involved in the control of the final effects of PO system activation was discovered and characterized in *T. molitor* [137]. The recombinant *T. molitor* 43 kDa protein decreased the melanin synthesis in vitro without influencing PO activity, indicating the possibility of controlling the course of the melanization reaction. The molecular mechanism regulating PO-induced melanin synthesis was studied in *T. molitor* by Kan et al. [93]. They provided biochemical evidence that, in this insect species, 79 kDa proPO and clip-domain SPH1 zymogen are converted by the Spätzle-processing enzyme to an active melanization complex consisting of the 76 kDa PO and clip-domain SPH1 active form. The melanization reaction initiated by this complex was tightly regulated by *Tenebrio* proPO, functioning as a competitive inhibitor of melanization complex formation [93]. Moreover, in *G. mellonella*, involvement of two hemolymph proteins, namely apoLp-III and Gm protein-24, in the regulation of the proPO cascade and PO activity was postulated [138]. Further research on *G. mellonella* indicated that lysozyme and some AMPs, namely *Galleria* defensin, proline-rich peptide 1, and anionic peptide 2, decreased PO activity in the hemolymph considerably [139]. A role of insect lysozymes as factors controlling the PO system and melanization was reported in *M. sexta*, where the lysozyme prevented conversion of proPO to PO by direct interaction with the proenzyme [140].

A role of functional amyloids in melanin deposition has also been reported [22,99,141]. For example, in *Heliotis virescens* hemocytes, an amyloidogenic P102 protein, which can form amyloid fibrils in the endoplasmic reticulum, is produced. Immune challenge induces release of these amyloid fibrils, which form an extracellular fibrillar scaffold and enhance melanin deposition on the surface of the intruder. A P102 homolog with characteristics similar to those of the *H. virescens* P102 protein was also identified in *Trichoplusia ni* [142]. Moreover, a lower level of the P102 protein in *A. mellifera* corresponded to decreased melanization and encapsulation in insects in the presence of deformed wing virus, i.e., a honey bee pathogen [143].

This complex network of molecules regulating the PO system and the PO itself indicates how important this system is for insect immunity. The control of endogenous PO activity is necessary for host self-protection against the cytotoxic action of reactive compounds generated during the melanization process and is extremely important for survival. For example, negative consequences of PO system activation were demonstrated in research on *T. molitor*, where melanization was associated with damage and impaired function of grafted Malpighian tubules (MTs) [144]. Moreover, it has been reported that the melanization response to *S. aureus* infection in young adult *T. molitor* can cause autoreactive tissue damage to MTs and faster aging. RNAi knockdown of PO transcripts led to partial rescue of MT function and an increased lifespan after infection [145]. An additional argument in this direction may stem from the results of studies conducted recently on *B. mori*, which documented the presence of a pair of melanized tubes (called PPO-positive tubes) protruding from the hindgut and strongly adhering to the MTs in larvae of this species [146]. ProPO was detected in epidermal cells inside these tubes. This indicates that MTs are not directly connected to the hindgut, and PPO-positive tubes constitute a connection between these organs, in which detoxification of metabolic wastes collected from hemolymph by MTs probably takes place. At the same time, this shows a way developed by this lepidopteran species to protect MTs from the dangerous effects of PO activity [146]. In previous research, a link between an overactive melanization response and a decreased survival rate was evidenced in *D. melanogaster* mutants in *spn27* encoding Serpin-27A required for specific inhibition of the proPO-activating enzyme, i.e., a terminal serine proteinase in a cascade [147]. On the other hand, silencing of *CG3066*-encoding serine protease Sp7, which is necessary for the activation of PPO1 [148], resulted in a slightly increased lifespan in comparison with the wild-type strain after infection with *Streptococcus pneumoniae*, but not with *Listeria monocytogenes* and *Salmonella typhimurium*, which significantly decreased the lifespan of infected flies, or with *Escherichia coli*, causing no change in killing rates in *CG3066* mutants [149]. The development of such a complex immune response, including the melanization response, implicates tuning of the response by different host–pathogen interactions during the evolution and coexistence of insects and their pathogens/parasites. Pathogens use the PO system and melanization for their own purposes (see Section 3), but the functions of this system for insect immunity are so essential that they are still maintained by insect organisms, despite being a source of molecules that are also dangerous to the host. This means that the benefits of this system are so important that evolution did not allow it to be eliminated, and therefore insects have developed complex ways of controlling the PO system [103].

## 3. Phenoloxidase System and Entomopathogenic Organisms

The picture presented above becomes even more complex when one takes into consideration the host–pathogen/parasite interactions and different strategies developed by intruders to suppress the host PO system and melanization. This complex network is also connected with genotype–genotype interactions between an insect host and an invader. The outcome of infection/infestation depends on the level of host susceptibility and on the capability of the pathogen/parasite to overcome the mechanisms of host immunity. Research conducted on *D. melanogaster* and a parasitoid wasp *Leptopilina boulardii* indicated that some aspects of immune response, i.e., activity of the PO system, are mainly affected by the host genotype, while others, e.g., differentiation of lamellocytes, depend on the combination of the host and parasite genotypes. However, on the basis of the host susceptibility to one genotype of the intruder, it is difficult to predict how sensitive this host will be to a different genotype of the same pathogen [150]. Interestingly, such host–pathogen/parasite interplay can also contribute to the development of host resistance. *D. melanogaster* exposed to intense parasitism by *L. boulardi* developed a more reactive cellular immune response connected with constitutive upregulation of immune-inducible genes responsible for the differentiation of lamellocyte precursors. The larvae evolved resistance based on cellular response, which changed from induced to constitutive [151]. Furthermore, *D. melanogaster* populations subjected to high parasitism pressure showed both more successful encapsulation and melanization of wasp eggs compared to insects that were not subjected to parasitism pressure [152]. This section will present selected strategies for exploiting the host PO system to evade immune mechanisms by entomopathogenic organisms.

### 3.1. Bacterial and Fungal Pathogens

Insect pathogenic bacteria are widespread and include members of, e.g., the genera *Bacillus*, *Lysinibacillus*, *Paenibacillus*, *Pseudomonas*, *Serratia*, *Yersinia*, *Photorhabdus*, and *Xenorhabdus*. Entomopathogenic bacteria (EPBs) usually infect their hosts through the oral route, penetrate the midgut epithelium using specific toxins, and eventually reach the hemocoel. However, some of them, e.g., *Serratia marcescens* and *Pseudomonas aeruginosa*, need to directly enter the hemocoel through the injured cuticle. On the other hand, *Photorhabdus* and *Xenorhabdus* are Gram-negative symbiotic bacteria of entomopathogenic nematodes from the genera *Heterorhabditis* and *Steinernema* (see the next subsection) [153,154].

The best-known and best-studied EPB is *Bacillus thuringiensis*, which has long been widely used to control insect pests in the orders Lepidoptera, Coleoptera, Diptera, Hymenoptera, and others. It is a spore-forming Gram-positive bacterium producing different types of toxins, i.e., crystal (Cry) proteins, synthesized as protoxins, cytolytic (Cyt) proteins, and vegetative insecticidal (Vip) proteins [155,156,157]. Cry proteins are produced by *B. thuringiensis* during sporulation and form crystalline inclusions called parasporal bodies, while Vip proteins are produced and secreted by vegetative bacterial cells in the host body. After ingestion by the insect host, the parasporal bodies are solubilized in the midgut and the Cry protoxins are activated by insect midgut proteases. Cry and Vip proteins are pore-forming toxins forming oligomers that are inserted into the membrane of midgut epithelial cells, leading to disruption of the midgut barrier, which allows gut microbiota (and *B. thuringiensis*) to enter the body cavity. The result is septicemia and death of the insect [155]. Ingestion of *B. thuringiensis* or its toxins leads to the activation of different host immune response mechanisms, including the synthesis of AMPs and activation of proPO in the midgut [158,159,160,161,162,163]. The melanization response in the midgut is important for resisting infections by *B. thuringiensis*, but it is also the target of manipulation by the pathogen. It was shown that infection of *Plutella xylostella* with the *B. thuringiensis* 8010 strain led to suppression of the *PPO1* gene and induction of four serpin genes in larval midgut, resulting in inhibition of melanization. Larval survival increased after feeding the infected larvae with recombinant proPOs [164]. Interestingly, the involvement of proPOs in fighting against *B. thuringiensis* and its toxins manifests itself not only by initiating the melanization process but also by binding bacterial toxin molecules, causing their inactivation, as shown in studies on *Ostrinia furnacalis* treated with the Cry1Ah toxin. In addition, the Cry1Ah-resistant *O. furnacalis* strain showed a 7.5-fold increase in *PPO* expression level in comparison to the susceptible strain [165]. Studies conducted in this area demonstrate that, at least in some insect species, there is an early activation of the melanization response after ingestion of *B. thuringiensis* and its toxins, and the increased level of the expression of proPO genes, as well as higher PO activity in resistant insects compared to susceptible ones, indicates an important role of this response in the interaction of the host insects with *B. thuringiensis*. The main and best-known way for insects to become resistant to *B. thuringiensis* is to modify the receptors responsible for initiating toxin binding to epithelial cell membranes. However, the relatively rapid acquisition of such resistance necessitates the search for other methods of controlling insect pests. Studies on the participation of the PO system and melanization response in the *B. thuringiensis*-host insect interaction provide a better understanding of these relationships and information that can be used in the development of new strategies for controlling insect pests.

Entomopathogenic fungi can attack a wide range of insect hosts and cause many insect diseases. They have evolved a number of strategies to counteract the immune defense of insects. In the fight against fungal infection, insects rely on avoidance behavior, physical barriers, and innate immune responses [166,167,168]. However, just as the insect’s immune system responds to the presence of a pathogen, the pathogen in the host’s body can sense the presence of host-produced antifungal molecules and trigger its own response against them. For example, during infection of *G. mellonella*, *Metarhizium robertsii* can respond by inducing the synthesis of chymotrypsin-like proteinases and metalloproteinases that degrade insect-derived antifungal peptides and proteinase inhibitors. The level of the response depended on the fungal strain and was epigenetically regulated to induce insect mortality after infection. For the development of this response, reciprocal communication using specific molecules was necessary during the co-evolution of the fungus and its insect host [169].

The first and most effective physical barrier preventing the entry of pathogens into the body is the cuticular layer; however, entomopathogenic fungi easily infect their hosts by direct penetration of the cuticle thanks to production of degrading enzymes, including proteases, chitinases, lipases, esterases, phospholipase C, and catalase [170,171,172,173,174]. The insects’ integumentary antifungal defense comprises, among others, activation of PO and melanin synthesis that limits the growth of some fungi and prevents the synthesis of cuticle-degrading enzymes [166,175,176]. Inside the insect cavity, the fungus switches to hyphal bodies, which are able to invade the entire insect body by a tissue-specific sequential process and finally produce insect mummification. They secrete toxic compounds, such as secondary metabolites that are crucial for survival and facilitate fungal invasion [177,178,179]. *Beauveria bassiana*, widely used in insect pest control, secretes different secondary metabolites, e.g., beauvericin, bassianin, bassianolide, beauverolides, oosporein, and oxalic acid [180,181,182]. Research on *G. mellonella* revealed that a *B. bassiana* bibenzoquinone, oosporein, was required for full fungal virulence and decreased PO activity in hemolymph, probably by blocking proPO cleavage [183]. Similarly, a group of cyclo-hexadepsipeptides called destruxins produced by different *Metarhizium* species can inhibit melanization response and suppress oxidative burst and antimicrobial peptide synthesis in infected insects [178,184,185]. Inhibition of melanization is beneficial for entomopathogenic fungi because it facilitates the evasion of this defense mechanism, allowing their growth and development in the host insect. As demonstrated by Wang et al. [184], the biosynthesis of destruxins in different *Metarhizium* species depends on the presence of the *dtxS1* gene, the acquisition and maintenance of which were correlated with evolution of host specificity of the fungus [184]. By enhancing the production of these secondary metabolites by the fungus in the infected organism or by their use thereof as additional components, the effectiveness of bioinsecticides based on *Metarhizium* spp. can be improved. In this context, it is important to indicate that *Beauveria* and *Metarhizium* species are the most frequently commercialized fungi for the control of insect pests which help to reduce the use of chemical pesticides. Moreover, transgenic *Metarhizium* spp. producing potent insect-selective toxins have been developed for the control of *Anopheles* mosquitos transmitting malaria parasites [186,187]. In the brown citrus aphid *Aphis citricola*, inhibition of melanization was caused indirectly by suppressing the scavenger receptor AcSR-B(CD36) by destruxin A. However, after *AcSR-B* silencing by feeding aphids with *dsAcSR-B*, no further reduction in PO activity by destruxin A was observed [188]. The possibility of using *dsAcSR-B* instead of destruxin A to obtain a similar final effect in aphids indicated another avenue for the development of dsRNA-based biopesticides for pest management based on RNAi technology [188]. Another strategy based on multifunctional laccase was described in *B. bassiana*. The extracellular laccase BbLac2 produced in the hyphal bodies during infection oxidized and deactivated PO and detoxified ROS generated during immune response in the insect hemocoel [189].

In the genomes of *Metarhizium* species, about 100 genes encoding different families of metalloproteinases have been identified [190]. Some of them have been shown to be important for fungal virulence, including MrM35-4, a member of the M35 family [191]. It has been evidenced that MrM35-4 is required for full virulence of *M. robertsii* against insect hosts. In a study on *D. melanogaster* and *G. mellonella*, it was demonstrated that MrM35-4 suppressed antifungal gene expression (*gallerimycin* in *G. mellonella* and *drosomycin* in *D. melanogaster*) and induced cell apoptosis. Moreover, it contributed to the inhibition of melanization in the insect cuticle and hemocyte-mediated melanization. MrM35-4 has been shown to cleave *D. melanogaster* recombinant PPO1 and PPO2 differently than proPO-activating serine proteases, indicating that the inhibition of melanization is related to the prevention of proPO activation to PO [192]. It can be proposed that the action of MrM35-4 represents yet another strategy to evade the host immune response by targeting specific pathways, including prevention of proPO activation. The molecular-level information provided by such studies can be used to develop insect pest control strategies based on targeting specific molecular pathways.

In some fungi, cell wall components recognized by insect PRRs as PAMPs can also play a role as fungal virulence factors affecting melanization. For example, *Aspergillus niger* α-1,3-glucan activated mechanisms of humoral and cellular immune response in *G. mellonella* [12,13]; however, temporary inhibition of PO activity in hemolymph after injection of *A. niger* α-1,3-glucan or conidia was observed [11]. Moreover, the study demonstrated a significant increase in the number of oenocytoids in the hemolymph of immunized *G. mellonella* larvae. As these hemocytes are responsible for proPO synthesis and release, it is possible that, in this host–pathogen interplay, the insect immune system ensures in this way an adequate level of PO activity for further melanization processes.

It is also worth noting here that yet another mechanism of melanization response suppression has been described in *Nosema bombycis* [193], a single-cell intracellular parasite belonging to microsporidia, previously considered to be related to fungi but now classified into the SAR-inclusive class or supergroup [194]. Namely, a serpin called NbSPN6 produced by this parasite can inhibit melanin formation in the hemolymph of its host, i.e., *B. mori*, by direct targeting the proPO-activating enzyme [193].

### 3.2. Entomopathogenic Nematodes and Their Symbiotic Bacteria

Entomopathogenic nematodes (EPNs) are a group of soil-dwelling nematodes that are capable of infecting a wide range of insects. Two genera of EPNs, i.e., *Steinernema* and *Heterorhabditis*, are used as biological agents against various insect pests and represent a promising alternative to pesticides. A key role in their pathogenicity against insects is played by endosymbiotic bacteria of the genera *Xenorhabdus* and *Photorhabdus*, respectively, which are vectored into insects by infective-stage juveniles. The free-living infective juveniles penetrate insect hosts through natural body openings or through spiracles and, 0.5 h–2 h after entering the host body, they release their symbiotic bacteria inside the hemocoel, where they develop and eventually kill the host by septicemia. By digesting host tissues, the bacteria provide a food source for the nematodes to mature and reproduce inside the host cadaver [195,196]. The endosymbionts also protect insect cadavers against competition from other microorganisms through the production of different antimicrobials. In turn, the nematode serves as a vector for the symbiotic bacteria and provides favorable conditions by interacting with the host immune system during the early phase of infection. This mutualistic relationship enables nematodes to exploit a broad array of insect hosts. On the other hand, the interaction between the insect hosts and EPNs is based on immunological recognition and insect immune response mechanisms. The main strategies that enable the survival and development of EPNs in the insect host are immune evasion and immunosuppression [197]. 

It has been demonstrated that the effectiveness of the nematode–bacteria complex in infecting host insects not only depends on the symbiotic bacteria but also can be attributed to the nematode itself. The nematode can actively impair the early immune response of the host [198,199]. Since proPO activation is initiated at the earliest after detection of an invader, it is important for the nematode to avoid recognition by the host immune system. To achieve this, EPNs can use molecular mimicry. Brivio et al. [198] showed that, after infection with both live and dead *Steinernema feltiae*, the activity of the PO system in *G. mellonella* larvae was significantly reduced shortly after infection, however before releasing endosymbionts. In addition, purified *S. feltiae* cuticle inhibited PO activity in vivo and in vitro, indicating an important role of the parasite’s body surface in the early phase of parasitism, possibly by interfering with host proPO activation pathways [198]. It was found that *S. feltiae* was able to modulate the activation of the *G. mellonella* PO system by removing various humoral factors from the hemolymph, which were specifically sequestered by the lipidic compounds of the *S. feltiae* epicuticle. In addition, the coating of the parasite with host hemolymph compounds contributed to its mimicry. Lipid extracts from the nematode cuticle inhibited PO activity in vitro and in vivo. Interestingly, the inhibitory effect was observed in the presence of the *S. feltiae* cuticle but not in the presence of the cuticle of the free-living *P. rigida* nematode [198,199].

In addition to the cuticular lipids of *S. feltiae*, host immune processes can be affected by different molecules excreted or secreted (excreted/secreted products, ESPs) by EPNs, e.g., serine proteinases, protease inhibitors, metalloproteases, and aspartic and cysteine proteases [200,201,202]. Research conducted by Balasubramanian et al. [203,204] revealed that two proteases secreted by *Steinernema carpocapsae* suppressed *G. mellonella* PO activity in vitro and in vivo, which contributed to avoidance of encapsulation by *G. mellonella* hemocytes. Increased expression of one of these proteases, a chymotrypsin-like protease, was observed in *S. carpocapsae* when infective juveniles were exposed to *G. mellonella* hemolymph, indicating that it acts as a virulence factor in this EPN [203,204]. Similarly, *G. mellonella* PO activity was inhibited by *Heterorhabditis bacteriophora* ESPs [205]. 

The insect host can protect itself from penetration by some EPNs by clot formation at the site of invasion. In the clot formation, phenoloxidase is involved, which is responsible for hardening and melanization of the clot matrix [206]. Research on *D. melanogaster* revealed that, in the case of *H. bacteriophora* invasion, the PO activity was restricted to the clot, while in the case of *S. carpocapsae*, it was enriched in the hemolymph. It was found that *S. carpocapsae* produces a specific serpin, Sc-spn6, which prevents the association of proPO with the clot. This impairs the formation of a mature clot, and therefore, the clot does not protect the insect from penetration by the nematode [207]. The role of all three *D. melanogaster* proPO genes, *PPO1-PPO3*, in the anti-*S. carpocapsae* immune response was investigated by Cooper et al. [208]. Using axenic and symbiotic nematodes, they demonstrated that the expression of all proPO genes contributed to host survival; however, only *PPO1* or *PPO3* were upregulated during the infection with the axenic or symbiotic nematodes, respectively. In addition, the infection with the axenic nematode resulted in higher levels of PO activity in the hemolymph than the infection with the symbiotic ones, indicating that *Xenorhabdus nematophila* can be responsible for suppression of PO activity in the *Steinernema*/*Xenorhabdus* complex [208]. More recently, Sanda et al. [209] reported downregulation of the expression of genes encoding components of the PO system in the Nipa palm hispid *Octodonta nipae* (Coleoptera) upon infections with *S. carpocapsae* and *H. bacteriophora*, including serine protease P56 (*SPP56*), proPO-activating factor 1 (*PPAF1*), PPO, and serpin 28 (*SPI28*) [209]. In a further study, when *O. nipae* were infected with EPN symbiotic bacteria, i.e., *X. nematophila* and *Photorhabdus luminescens*, these authors evidenced that the bacteria and their metabolites contributed significantly to downregulation of these genes. In addition, both live endosymbionts inhibited PO activity in *O. nipae*, unlike heat-killed bacteria, which strongly increased PO activity [210].

The role of EPN endosymbiotic bacteria *P. luminescens* and *X. nematophila* in EPN parasitism is connected, among others, with the metabolites they secrete, which were reported to inhibit the activation of the insect PO system responsible for melanin production. For example, both bacteria are producers of rhabduscin, an amidoglycosyl- and vinyl-isonitrile-functionalized tyrosine derivative able to inhibit PO activity [211]. They can also disrupt the functioning of the PO system by generating small-molecule antibiotics called hydroxystilbenes. *P. luminescens* produces (E)-1,3-dihydroxy-2-(isopropyl)-5-(2-phenylethenyl)benzene (ST), the synthesis of which requires the enzyme encoded by the *stlA* gene. A *Photorhabdus stlA^-^* mutant was significantly less virulent and provoked the formation of considerably more melanotic nodules in *M. sexta* than wild-type bacteria. It was shown that inhibition of PO activity by ST is a crucial factor that mediates these effects of *Photorhabdus* infection [212,213].

It is known that eicosanoids play an important role in the cellular and humoral immune response in insects. Dysregulation of eicosanoid synthesis can lead to suppression of different immune mechanisms, including PO system activity. Inhibition of PO activation can be the result of eicosanoid biosynthesis inhibition *via* suppression of phospholipase A2 (PLA2) [214,215]. Eicosanoid biosynthesis can be inhibited by a nematode–bacterium complex because, among metabolites secreted by endosymbionts during infection, there are molecules able to inhibit insect PLA2, e.g., oxindole and benzylideneacetone (BZA) [216]. This effect was observed in *Spodoptera exigua* after infection with *S. carpocapsae*/*X. nematophilus*. In addition to PLA2 inhibition, BZA can directly inhibit the PO activity by suppression of proPO activation [217,218,219].

In addition to inhibiting the activity of the PO system, especially at an early phase of nematode–bacterium complex invasion, endosymbionts can affect host survival by causing excessive activation of this system and melanization. For example, *Xenorhabdus budapestensis* was found to produce a HIP57 toxin with homology to the GroEL chaperone, which caused blackening of *G. mellonella* larval bodies by activation the PO cascade and was potentially responsible for larval death [220]. Similarly, when injected into *G. mellonella* larvae, *Xenorhabdus ehlersii* GroEL activated proPO and reduced the number of hemocytes, which led to the death of insects within 48 h [221]. Moreover, the recombinant GroEL of *X. nematophila* SC 0516 also worked by overactivating proPO in *G. mellonella* larvae [222].

**Figure 5:**
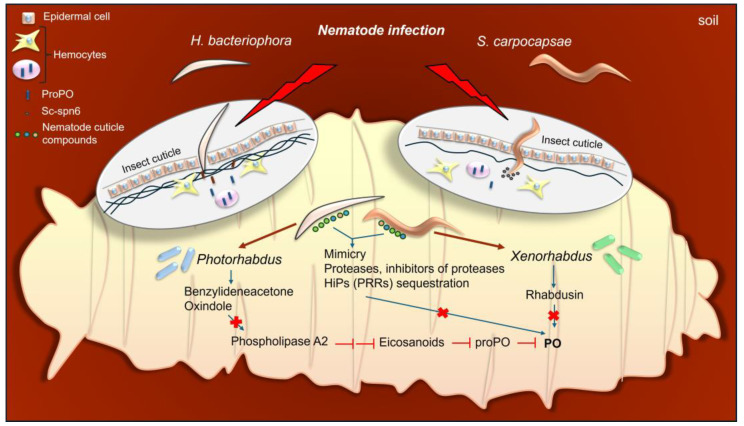
Suppression of PO activity by *Heterorhabditis*/*Photorhabdus* and *Steinernema/Xenorhabdus* nematode–bacterial complexes in an insect larva. Further details can be found in the text.

The above brief presentation of the ENP-symbiotic bacteria–insect host relationships clearly shows that each of the participants takes an active part in this relationship, e.g., by influencing the functioning of the host PO system. This shows the importance of this system and the melanization process in insect immunity. These relationships are schematically presented in Figure 5.

### 3.3. Parasitoid Wasps and Their Armament

During parasitism, female parasitoid wasps (Hymenoptera) lay eggs inside (endoparasitoids) or outside (ectoparasitoids) the insect host and at the same time introduce factors that facilitate successful development of the parasitoid larva. Parasitoid wasps have evolved various strategies to ensure successful parasitism, including the introduction of venom components, teratocytes, polydnaviruses (PDVs), and virus-like particles (VLPs) into the insect body, in order to modulate the host immune response and metabolic homeostasis, benefiting the survival of wasp offspring by disabling host cellular and humoral immune defenses [191,223,224,225]. Since the mechanism of encapsulation and killing of parasitoid eggs by melanization is common, the inhibition of melanization by parasitoids is particularly important for their survival in the insect host.

#### 3.3.1. Venom Components

Wasp venom has been shown to be a cocktail of protein and non-protein components that make it possible to control the development of insect hosts or lead to disorders in their health. Wasp venom proteins can manipulate the host immunity to avoid detection of the parasitoid by the host immune system. Serine proteases (SPs), serine protease homologs (SPHs), and different types of serine protease inhibitors (SPNs), which can modulate host organisms, have been identified in parasitoid venoms [224,226].

*Pteromalus puparum* (Hymenoptera, Pteromalidae) is a generalist endoparasitoid wasp that parasitizes the pupal stage of several lepidopteran species, including the small cabbage white butterfly, *Pieris rapae*, an agricultural pest. The *P. puparum* genome encodes 145 SPs and 38 SPHs. Some SPs/SPHs are expressed in venom glands, suggesting their specific physiological functions as venom proteins [227,228,229]. In *P. puparum* venom, serpin PpS1V encoded by the *PpSerpin-1* gene was detected. It was shown that alternative splicing increases PpSerpin-1 protein diversity and several isoforms can be recruited to wasp venom to suppress host immunity. Sixteen predicted PpSerpin-1 splicing isoforms differ only in the C-terminal region. It was shown that the recombinant PpSerpin-1 isoform identified in *P. puparum* venom (rPpS1V) inhibited in vitro activation of host proPO, rather than directly inhibiting PO activity. Moreover, rPpS1V formed complexes with two hemolymph proteins, i.e., *P. rapae* hemolymph proteinase 8 (PrHP8) and *P. rapae* prophenoloxidase-activating proteinase 1 (PrPAP1) [229]. It was also observed that rPpS1V inhibited activation of proPO of a non-natural host, the Asian corn borer *O. furnacalis,* via interaction with OfSP13, an ortholog to PrPAP1 [228]. Alternative splicing leading to increased serpin diversity was also described in other parasitoid wasps, e.g., *Copidosoma floridanum*, *Trichogramma pretiosum*, and *Orussus abietinus* [228,230].

In the venom of *Microplitis mediator* (Hymenoptera: Braconidae), inhibitors of serine proteases from the serpin superfamily, which interact with the host immune system through serine proteases, were identified. *M. mediator* venom serpins MmvSPN-1 and MmvSPN-2 regulate the humoral immune response in two lepidopteran hosts, the cotton bollworm *Helicoverpa armigera* and the oriental armyworm *Mythimna* (*Pseudaletia*) *separata*, affecting the PO system and the expression of AMP genes. It has been demonstrated that recombinant MmvSPN-1 and MmvSPN-2 suppressed host melanization by binding to HacSP29, a clip-domain serine protease that controls proPO activation [231].

In addition to inhibitors belonging to the serpin superfamily, Kazal-type serine protease inhibitors have been characterized in venoms, e.g., an inhibitor named PvKazal in the venom of the drosophilid ectoparasitoid *Pachycrepoideus vindemmiae* (Hymenoptera: Pteromalidae). Recombinant PvKazal inhibited melanization of *D. melanogaster* hemolymph. Furthermore, it was shown that heterologous expression of PvKazal in transgenic *Drosophila* decreased the number of crystal cells and blocked melanization of the host hemolymph [232,233]. Two Kazal-type serine proteinase inhibitors (NvKSPI-1 and -2) identified in the venom of the generalist parasitoid *Nasonia vitripennis* (Hymenoptera: Pteromalidae) suppressed proPO activation in the hemolymph of the *M. domestica* host [234].

*Cotesia rubecula* (Hymenoptera: Braconidae) negatively regulated the activation of the host proPO by a venom 50 kDa serine proteinase homolog (SPH) designated as Vn50. It was found that recombinant Vn50 can bind components of *P. rapae* hemolymph, i.e., proPO and proPO-activating proteinase (PAP). Moreover, Vn50 effectively reduced proPO activation via *M. sexta* PAP-1, SPH-1, and SPH-2 but did not inhibit active PO or PAP-1 [235]. Similarly, a serine proteinase homolog, SguaSPH, abundant in the venom of the ant-like bethylid ectoparasitoid *Scleroderma guani* (Hymenoptera: Bethylidae), can inhibit melanization in the coleopteran species *T. molitor* and PO activity in the lepidopteran species *O. furnacalis* [236].

Research on the parasitoid wasp *Aphidius ervi* (Hymenoptera: Braconidae) and its pea aphid *Acyrthosiphon pisum* host revealed that injection of the wasp venom enhanced the host mummification, changed gene expression, and decreased PO activity in *A. pisum* [226]. It was suggested that the serine protease homolog 1 (*Ae*SPH1) and the serine protease inhibitor (*Ae*SPN1) identified in the *A. ervi* venom gland regulated PO activity in the host. It was found that the *A. pisum* injection with recombinant *Ae*SPH1 and *Ae*SPN1 effectively suppressed melanization and selectively reduced the expression of *ApPPO1*, whereas the injection of *A. ervi* venom into the *A. pisum* hemocoel resulted in significant upregulation and downregulation of *ApPPOs* gene expression at 6 h and 48 h after treatment, respectively [226]. The results indicated that the initially increased expression of *ApPPOs* induced by the venom was part of the host immune response aimed at protection against the invader. Then, the venom proteins suppressed this host response, leading to reduced melanization to protect the development of parasitoid eggs. The essential role of PO in protecting the host against parasitoid wasp is initiation of melanin deposition on the surface of the egg during the encapsulation process. The melanin layer finally prevents hatching of the egg and thus protects the host against parasitoid larva development. Because the suppression of melanization response by the venom proteins (and other factors) enables egg hatching, it simultaneously increases the likelihood of further development of the larva in the host’s body, especially since the parasitoid larva can also manipulate host physiology, including further suppression of melanization, e.g., by proteins secreted by teratocytes [237]. Analysis of venom components and their functions is consistent with the “RNAi plus” strategy of pest management that combines parasitoid wasps with dsRNA-targeting genes encoding venom-interacting proteins in specific hosts [226,238].

#### 3.3.2. Polydnaviruses and Virus-like Particles

Endoparasitoid wasps, which lay their eggs inside the bodies of other insects, have acquired symbiosis with viruses, which enable the parasitoid larvae to develop inside other organisms and maximize the success of their parasitic lifestyle. The symbiosis has evolved into the integration of the full virus genome into the genome of the parasitoid. Polydnaviruses (PDVs) constitute a special type of large double-stranded DNA viruses in parasitic insects that do not replicate in infected hosts. PDVs are classified into two different genera: bracovirus (BV) and ichnovirus (IV), i.e., symbiotic viruses of braconid and ichneumonid parasitoid wasps, respectively [182,223,239].

In the parasitic wasp *Microplitis demolitor* (Hymenoptera: Braconidae), the bracovirus (MdBV) carried by the wasp encodes Egf proteins, with inhibitory activity toward the PO system. It was shown that the three-member family of MdBV *Egf* genes encodes the only factors responsible for suppressing insect melanization. Egf proteins belong to a family of proteins that share a common cysteine-rich motif with similarities to the trypsin inhibitor-like (TIL) domains of small serine proteinase inhibitors (smapins). Egf1.0 mutant constructs indicated that Egf1.0 blocks proPO activation via PAP inhibition. Experiments with wild-type Egf1.0 showed inhibition of *M. sexta* PAP1 and PAP3. Moreover, a further study revealed that Egf1.0 paralog, Egf1.5, also bound PAP1, PAP3, and SPH2 and inhibited the activity of *M. sexta* PAP1 and PAP3. Egf1.5 and Egf1.0 similarly inhibited melanization in four lepidopteran species: *Pseudoplusia includens*, *Helicoverpa zea*, *M. sexta*, and *B. mori* [240,241].

**Figure 6:**
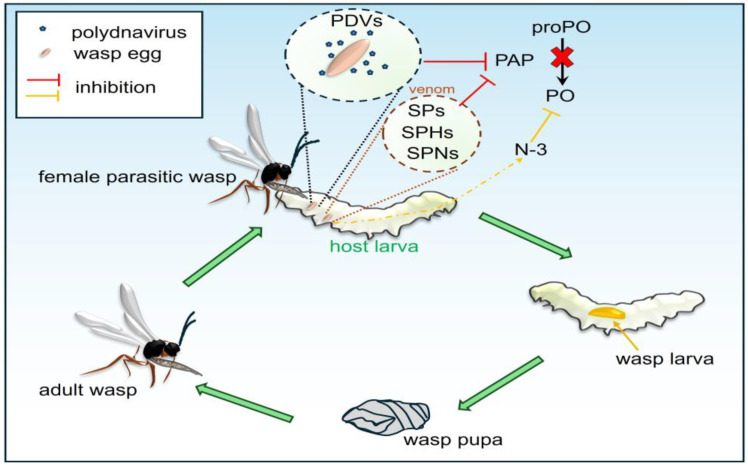
Simplified diagram of the life cycle of a parasitoid wasp using a lepidopteran larva as a host. Parasitoid wasps use various strategies to weaken the host’s immune system, e.g., during oviposition, females introduce factors that inhibit melanization in the host body, including polydnaviruses (PDVs), venom components, or nasonin-3 (N-3). This prevents the egg from being recognized by the host’s immune system. In this way, the larva can hatch and develop undisturbed, feeding on the corpse of the infected insect. PDVs suppress the host immune responses after injection into insect host during oviposition along with egg(s) and other factors. PDV DNA enters the wasp cell nuclei and integrates into its genome; then, the expression of virulence genes occurs, which inhibits the host immune response and alters host development. Wasp venom contains serine proteases (SPs), serine protease homologs (SPHs), and serine protease inhibitors (SPNs). PDVs and venom components inhibit the activation of the proPO via PAP inhibition. Nasonin-3 is a defensin-like peptide from *Nasonia vitripennis* which can decrease PO activity [243].

When laying eggs, *Cotesia kariyai* (Hymenoptera: Braconidae) introduces PDVs and venom into the host body, which synergistically inhibit the host immune response. The *C. kariyai* polydnavirus (CkPDV) and the venom gland express a C-type lectin called Cky811 with high homology to a C-type lectin named Mys-IML of host *M. separata* larvae, which plays the role of a PRR. Hemocytes called hyperspread cells (HSCs) are the starting point of *M. separata* melanization response to *C. kariyai* parasitism. Sawa et al. [242] showed that, after injection of CkPDV and venom, HSCs could not adhere to *C. kariyai* larvae implanted in the host, which prevented encapsulation and melanization. It appeared that the injection of CkPDV and venom led to suppression of *Mys-IML* expression, while the Cky811 protein expressed in the host hemocytes affected the recognition of non-self, thereby inhibiting HSC adhesion, encapsulation, and melanization [242] (Figure 6).

Virus-like particles (VLPs) are ovoid-to-round-shaped, single-membrane vesicles ranging from 50 to 100 nm in size. They contain venom proteins and are stored in the venom reservoir. After injection into host larvae, VLPs target hemocytes and, once they enter the hemocytes, the packed venom proteins are released. *Leptopilina heterotoma* (Hymenoptera: Figitidae) can parasitize a range of different host species, mainly in the genus *Drosophila*. *L. heterotoma* produces 300 nm spiked VLPs and introduces them into the hemolymph of *Drosophila* larvae during oviposition. VLPs can bind to host’s hemocytes via surface projections. VLPs act by lysing lamellocytes and disrupting the generation of new lamellocytes through lysing the host lymph glands. This prevents encapsulation of *L. heterotoma* larvae by host lamellocytes, thus allowing successful development [244].

#### 3.3.3. Teratocytes

Teratocytes are specialized cells that dissociate from the embryonic serosal membrane of some endoparasitoid wasps during parasitism. They are released into the host insect body when wasp eggs hatch to regulate host homeostasis and provide a favorable environment for offspring development [225,245]. *Cotesia vestalis* (Hymenoptera: Braconodae) teratocytes release different inhibitors of serine proteinases, e.g., trypsin inhibitor-like CvT-TIL, which potently inhibits the activation of proPO in the diamondback moth *P. xylostella* hemolymph by interacting with PxPAP3 of the PO system [246]. Moreover, *C. vestalis* teratocytes secrete serpins called CvT-serpins. It was found that CvT-serpins 1, 16, 18, and 21 play a role in the inhibition of melanization. In addition, it was revealed that CvT-serpin 6 inhibited proPO activation by forming SDS-stable complexes with activated PxPAP1 and PxPAP3. The phylogenetic analysis showed that CvT-serpin 6 was clustered into one clade with one splicing isoform of *P. puparum* serpin, serpin 10, and *A. gambiae* Ag-serpin6 [247,248].

#### 3.3.4. Epigenetic Mechanisms

Epigenetic mechanisms regulate gene expression in eukaryotes, including insects. Major components of epigenetic regulatory machinery are DNA methylation, histone modifications, noncoding RNA profiling, and chromatin condensation and relaxation [249,250,251,252,253]. For example, histone reversible acetylation, which modifies chromatin structure and alters the accessibility of DNA to transcription factors, has been shown to regulate epigenetic transcriptional reprogramming during metamorphosis following wounding and during infection by pathogenic bacterium *Listeria monocytogenes* and entomopathogenic fungus *Metarhizium anisopliae* in a *G. mellonella* model host [253,254]. Another study reported on the role of miRNAs in the epigenetic reprograming of innate immunity in *G. mellonella* larvae to distinguish between pathogenic and commensal strains of *E. coli* [255].

There is evidence indicating that parasitoid wasps, especially those lacking PDVs or teratocytes, may influence host gene expression via epigenetic regulation through post-translational modification involving reversible acetylation of lysine residues in histones by histone acetyltransferase and histone deacetylase. *Tetrastichus brontispae* (Hymenoptera: Eulophidae) wasps use this mechanism to manipulate immune response in their host beetle *O. nipae* (Coleoptera). During parasitism, reduced expression of histone deacetylase Rpd3 and increased levels of lysine acetylation in histone H3.3 were observed. Interestingly, *Rpd3* knockdown resulted in increased expression of PO system components, i.e., *OnPPAF1* and *OnPPO*, which led to melanization of pupae, resulting in black clumps resembling mummified parasitized pupae [256,257]. Another sophisticated mechanism has been described in parasitoid wasps *Snellenius manilae* and *C. vestalis*, which influences genes crucial for the development of their hosts (*Spodoptera litura* and *P. xylostella*, respectively) by microRNAs derived from teratocytes and PDVs [258,259].

## 4. Phenoloxidase System from the Evolutionary Perspective

In addition to its essential role in the immune response, phenoloxidase is also involved in a number of different processes in the insect organism, e.g., wound healing, cuticle sclerotization, regulation of microbiota, antioxidant reactions, and oxygen transport, and can influence insect neuronal activity and development [31,33,61]. It can therefore be assumed that this diversity of functions will be reflected in the diversity of genes encoding the components of the PO system.

The phylogenetic analysis of insect proPO genes (75 genes) conducted by Lu et al. [33] revealed the phylogenetic tree with three major clades of insect proPOs: Clade A, containing conserved proPOs present in various insect orders, and Clade B and C, representing distinctive paralogs occurring only in Lepidoptera and Diptera, respectively [33]. As mentioned in Section 2.1, although different insect species differ in the number of genes encoding proPOs, one, two, or three proPO-encoding genes have been identified in most species. So far, the largest number of genes for proPO has been found in various species of mosquitoes (Diptera). Since the role of individual proPOs has not yet been fully understood, it is also difficult to clearly explain the reasons for such a large number of proPO genes in these insects.

An important process in evolution is gene duplication, which can result in the formation of large gene families. An effect of this process may be the separation of functions, whereby each gene takes over some of the existing functions of the parent gene, but also the acquisition of a new function. The research conducted by Dudzic et al. [32] suggested that, of the three proPO genes in *D. melanogaster*, *PPO3* emerged as a result of duplication of the ancestral *PPO2*-like gene in the lineage ancestor of *Drosophila*, which gave rise to the *D. melanogaster* subgroup, because most other *Drosophila* species possess two proPO genes, i.e., *PPO1* and *PPO2*. It is plausible that this duplication was connected with the development of encapsulation as a cellular immune response caused by the selective pressure of parasitoid wasps. Further diversification led to a state in which crystal cells and lamellocytes express *PPO2* and *PPO3*, respectively [32].

However, mosquitoes constitute a special group of insects because a number of species are vectors of pathogens that cause dangerous diseases in humans. Unlike in *D. melanogaster*, the proPO genes in mosquitoes constitute a gene family that arose through gene expansion. The analysis and comparison of immune signaling pathways and response modules in *A. aegypti* (a yellow fever and Dengue vector), *A. gambiae* (a malaria vector), and *D. melanogaster* conducted by Waterhouse et al. [260] revealed different dynamics of functional gene categories and particular aspects of immune processes, indicating rapid evolution of some of them. In the melanization module, SPs and SPHs represent mosquito-specific expansions with no orthologs in *D. melanogaster*. Moreover, among proPOs, only *A. gambiae* PPO1 and *A. aegypti* PPO6 have orthologs in *D. melanogaster*, while the other 17 mosquito proPOs analyzed form a distinct clade that arose from reduplication events. The authors postulated that such expansion may reflect differential adaptation to different developmental challenges during evolution, including the need to interact with different transmitted parasites and pathogens [260]. In *A. gambiae*–*Plasmodium* interactions, melanization is regulated, among other ways, by a complex network of mosquito SPHs acting as activators and inhibitors of this defense mechanism [261]. It was demonstrated that melanization in refractory *A. gambiae* strains is involved especially in clearance of killed ookinetes, while melanization induced in susceptible strains by genetic manipulations is also engaged in the killing of the parasites. A more recent study has revealed that two of the nine proPOs in *A. gambiae*, i.e., PPO1 and PPO3, are the most involved in the anti-*Plasmodium* immune response that promotes oocyst killing, further confirming the important role of host–pathogen interactions during co-evolution in the diversification of proPO functions in different insect species [70]. To do so, they performed a detailed quantitative evolutionary feature profiling of genes and gene families involved in innate immunity in 68 insect species, including 22 mosquito species [262]. The melanization modulator–effector pair of serpins and proPOs showed the highest expression similarity but with negligible evolutionary similarity, suggesting that the regulation and execution of these responses are subject to different constraints during evolution [262].

On the basis of phylogenetic analysis of proPOs across all genera of mosquitoes and other insects, Zhu et al. [263] divided proPOs into two clades: (i) the classical insect type, including enzymes with oxidase activity (oxidation of *o*-diphenols to *o*-quinones), and (ii) the dipteran type, including those that exhibit both oxygenase (conversion of monophenols to *o*-diphenols) and oxidase activities. It was found that, while the first clade included proPOs from various insect species, the majority of the second clade consisted of proPOs described in dipteran species, mainly in mosquitoes. Interestingly, in each of the four mosquito species (*A. aegypti*, *A. albopictus*, *A. gambiae*, and *C. quinquefasciatus*), only one of the proPOs is an ortholog of proPOs of other insect species and belongs to the classical insect-type proPOs. Further detailed biochemical and physiological research on two proPOs of *A. aegypti*, i.e., PPO6 and PPO10, representing the classical insect type and the dipteran type, respectively, revealed different substrate specificities and roles of these proPOs. Homozygous *PPO6* knockout mutants were unable to grow beyond the first instar larval stage, indicating the essential role of PPO6 in *A. aegypti* larval growth and survival. On the other hand, *PPO10* knockout mutants exhibited abnormal body size, structural changes in the cuticle, prolonged development, and decreased immune response, suggesting a PPO10 impact on some biological processes [263].

## 5. Conclusions and Future Perspectives

Melanogenesis and melanin deposition are processes essential for the effective immune response of insects to various types of invaders. They provide different cytotoxic molecules active in fighting infections, as well as melanin, which is important for sequestration of pathogens and parasites. Proper activation of these mechanisms requires appropriate recognition of PAMPs and DAMPs mediating the activation of dedicated serine proteinase cascades, leading finally to the activation of proPO to its enzymatically active form. As indicated in the previous sections, the activity of the PO system is strictly regulated at many levels to minimize the harmful effects of cytotoxic products generated during the melanogenesis process on host cells. Studies of the melanization process in various insect species have shown that, despite the similar general course of activation, the PO system exhibits differences in the number of serine proteases, serine protease homologs, and serine protease inhibitors involved. Moreover, additional factors, e.g., SPHs, may be necessary for the activation of proPO to PO by a proPO-activating protease. Another aspect of diversity is the different number of proPOs found in different insect species and the involvement of different hemocyte subpopulations in their synthesis. Moreover, the activation of the PO system may proceed through different pathways, depending on the recognized stimulus (e.g., septic and aseptic injury), and may also partially involve components of serine protease cascades, leading to the activation of signal transduction pathways (e.g., the Toll pathway). The activation of the PO system can also be induced by proteases produced by infecting pathogens, which can lead to uncontrolled increased melanization, which can pose a risk of host death.

Such great diversity in the PO system of different insect species may indicate that this system was shaped through contacts of the host insect with various pathogens or parasites. This may reflect a history of co-evolution of host–pathogen/parasite interactions and the constant need to adapt the PO system to different factors threatening insects in their environment. As shown in the selected examples, pathogens/parasites can use different strategies to avoid or overcome insect immune mechanisms, including the ability to control the activity of the host PO system and the melanization process, which not only allows them to survive and develop in the host body but also sometimes even protects the intruders against other mechanisms of insect immune response.

Of note, different insect species are vectors that carry various pathogens that are dangerous to humans and other vertebrates. Therefore, understanding these strategies and mechanisms of manipulating the immune response of their hosts can help to better control the spread of diseases such as Chagas disease caused by *Trypanosoma cruzi*, which is transmitted by triatomines (Hemiptera: Reduviidae) [264,265,266], or viral diseases such as dengue fever, West Nile fever, and chikungunya fever, transmitted by *Aedes albopictus* and *Aedes aegypti* mosquitoes [267,268]. This issue is especially important in the face of ongoing climate change, which allows insect vectors to occupy areas where they have not been previously encountered, highly increasing the risk of spreading such diseases. Insects also serve as vectors of plant pathogens, including economically important plant rhabdoviruses. Many plant viruses use various counter-strategies to control the insect immune defense to promote virus survival in insect vectors. Some of them, e.g., rice stripe mosaic virus (RSMV) transmitted by *Recilia dorsalis* and *Nephotettix virescens* leafhoppers, evade the antiviral melanization response of the insect vector by interfering with the proteolytic cleavage of proPOs. Deeper knowledge of these mechanisms may lead to novel strategies for blocking the transmission of insect-borne plant viruses [269,270].

To date, studies of the insect host–pathogen/parasite relationship have provided detailed knowledge of the mechanisms used by pathogens and parasites to control the immune processes of the insect host. Thanks to this, both entomopathogenic fungi, e.g., *B. bassiana*, and parasitoid wasps are used as biological control agents against insect pests. The knowledge of these often sophisticated adaptive strategies and the availability of new technologies contribute to the development of more effective methods of controlling insect pests. An example of such an approach is the increase in *B. bassiana* virulence against *Helicoverpa armigera* and *Spodoptera frugiperda* achieved by introducing a gene encoding the VRF1 protein, which is a component of the venom of the parasitoid wasp *Microplitis mediator*, into the genome of the fungus. The VRF1 protein is a metalloproteinase that prevents the recruitment of hemocytes during encapsulation [271]. The available knowledge and the results of ongoing research facilitate the development of genetically modified bioinsecticides with higher efficacy and a broader spectrum of action. Moreover, the discovery of new, previously undescribed strategies for subordinating host immune mechanisms by various pathogens and parasites, including mechanisms of epigenetic regulation of gene expression and microRNA-based regulation of genes important for host development [254,258,259], not only provides a deeper understanding of complex host–pathogen relationships but also opens up new possibilities for the development of preparations for the biological control of pest insects or insect vectors.

## Data Availability

Not applicable.
